# Lung adenocarcinoma and lung squamous cell carcinoma cancer classification, biomarker identification, and gene expression analysis using overlapping feature selection methods

**DOI:** 10.1038/s41598-021-92725-8

**Published:** 2021-06-25

**Authors:** Joe W. Chen, Joseph Dhahbi

**Affiliations:** California University of Science and Medicine, Colton, CA USA

**Keywords:** Medical genomics, Genetic markers

## Abstract

Lung cancer is one of the deadliest cancers in the world. Two of the most common subtypes, lung adenocarcinoma (LUAD) and lung squamous cell carcinoma (LUSC), have drastically different biological signatures, yet they are often treated similarly and classified together as non-small cell lung cancer (NSCLC). LUAD and LUSC biomarkers are scarce, and their distinct biological mechanisms have yet to be elucidated. To detect biologically relevant markers, many studies have attempted to improve traditional machine learning algorithms or develop novel algorithms for biomarker discovery. However, few have used overlapping machine learning or feature selection methods for cancer classification, biomarker identification, or gene expression analysis. This study proposes to use overlapping traditional feature selection or feature reduction techniques for cancer classification and biomarker discovery. The genes selected by the overlapping method were then verified using random forest. The classification statistics of the overlapping method were compared to those of the traditional feature selection methods. The identified biomarkers were validated in an external dataset using AUC and ROC analysis. Gene expression analysis was then performed to further investigate biological differences between LUAD and LUSC. Overall, our method achieved classification results comparable to, if not better than, the traditional algorithms. It also identified multiple known biomarkers, and five potentially novel biomarkers with high discriminating values between LUAD and LUSC. Many of the biomarkers also exhibit significant prognostic potential, particularly in LUAD. Our study also unraveled distinct biological pathways between LUAD and LUSC.

## Introduction

Lung cancer is the most commonly diagnosed malignant tumor and is a leading cause of cancer-associated mortality. It is the second highest cause of new cancer cases in both genders in the United States and is the second leading cause of cancer deaths in females globally^1,2^. The most common subtypes of lung cancers are lung adenocarcinoma (LUAD) and lung squamous cell carcinoma (LUSC), classified together as non-small cell lung cancer (NSCLC)^3,4^. However, recent studies have suggested that LUAD and LUSC should be classified and treated as different cancers^5^.

Identifying the mechanisms underlying LUAD and LUSC is needed to develop useful biomarkers for better diagnosis and design therapeutic interventions. Multiple gene expression and immunohistochemistry studies have identified biological pathways and biomarkers that differentiate between LUAD and LUSC^6–8^. Other studies classified cancers using both novel and traditional machine learning or feature selection methods^9–12^. However, few have investigated cancers by applying multiple feature selection methods and selecting the overlapping features.

In this study, we downloaded LUAD and LUSC RNA-Seq datasets from The Cancer Genome Atlas (TCGA)^13^ and analyzed them with five feature selection methods with ranking abilities: Differential Gene Expression Analysis (DGE), Principal Component Analysis (PCA), Least absolute shrinkage and selection operator (Lasso), minimal-Redundancy-Maximal Relevance (mRMR), and Extreme Gradient boosting (XGboost). DGE applies a normalization method and uses the negative binomial distribution to detect significant changes in gene expression across samples^14,15^. Many studies have shown that DGE, though being the most widely used algorithm to detect differentially expressed genes, often yields some false positive results; in addition, it is often sensitive to outliers^14–17^. On the other hand, XGboost is a tree-based machine learning method that is not sensitive to outliers but is prone to overfitting^17,18^. To minimize this problem, we chose to use Lasso, a linear regression technique that avoids overfitting but can be influenced by highly correlated features and potentially leading to false discoveries^17–20^. mRMR is then used to maximize the relevance between the features and the output, and minimize the relevance among the feature themselves, thus, limiting highly correlated features^21–23^. PCA is another well-known and widely used feature reduction technique in machine learning to reduce high dimensional data into orthogonal principal components, which also removes correlated features^17,18^. However, amidst other disadvantages, the result of PCA by itself is often not interpretable^17,18^. These algorithms were also chosen because of their ability to rank features or select a reasonable number of features. In short, overlapping these algorithms is promising because different methods select features using different criteria. Since each method has its strengths and weaknesses, focusing on the overlapping features will optimize the strengths and minimize the weaknesses of each method, thereby reducing the number of false positives and producing reliable results. This study will serve as a proof of concept for the validity of the approach to overlap feature selection methods while investigating NSCLC subtype differences and discovering novel biomarkers.

## Results

### Study design and overview

**Figure 1:**
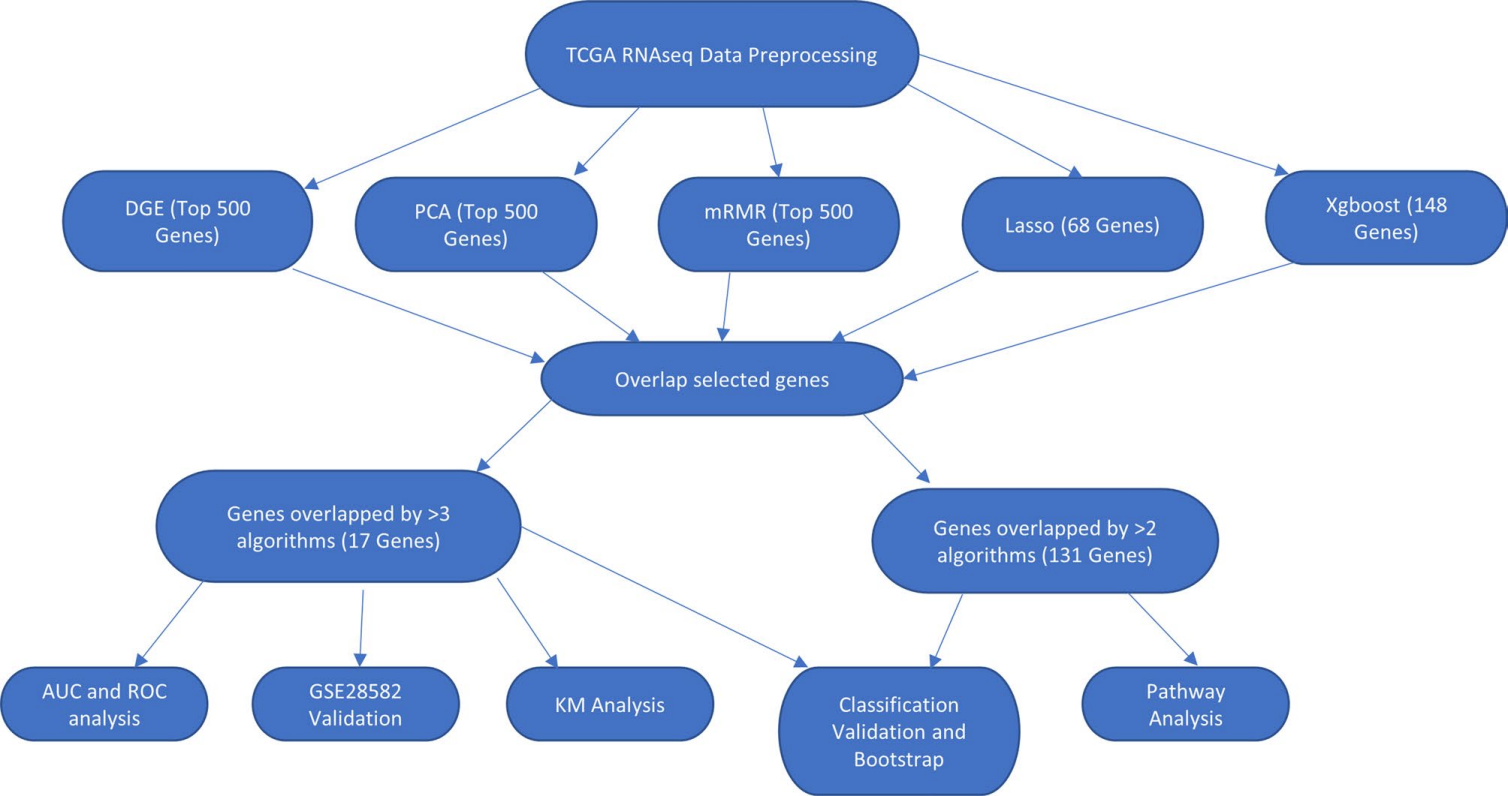
An overview of the experimental design. A scheme summarizes the selection methods and the numbers of the resulting overlapped genes.

We obtained LUAD and LUSC RNA-Seq data from TCGA^13^ and the summary of their clinical information was provided in Table 1, with more comprehensive details available on TCGA website^13^. We selected discriminatory genes by overlapping DGE, PCA, mRMR, XGboost, and lasso as depicted in Fig. 1. The genes that were overlapped by two or more algorithms were validated and used for LUAD and LUSC classification as well as gene expression analysis. The genes that were overlapped by three or more algorithms were selected as biomarker candidates, and their diagnostic values were assessed using ROC analysis and AUC value, and then further verified in an external dataset, GSE28582^24,25^, which is a microarray dataset that includes 50 LUAD and 28 LUSC samples The prognostic values of the biomarker candidates were also assessed using Kaplan Meier Plotter^26^.

**Table 1:** Summary of clinical information from TCGA with each entry indicating number of samples.

Gender		AJCC pathologic stage		Treatment		Primary diagnosis subtypes	
**Lung adenocarcinoma**							
Male	220	Stage IA	124	Pharmacotherapy only	56	Adenocarcinoma, NOS	311
Female	259	Stage IB	131	Radiotherapy only	101	Adenocarcinoma with mixed subtypes	108
Missing	50	Stage IIA	46	Both therapies	70	Papillary	22
		Stage IIB	63	No treatment	242	Bronchiolo-alveolar, NOS	3
		Stage IIIA	66	Missing	60	Bronchiolo-alveolar, nonmucinous	19
		Stage IIIB	11			Brionchio-alviolar Carcinoma, mucinous	5
		Stage IV	24			Micropapillary	3
		Stage I	5			Clear cell	2
		Stage II	1			Solid carcinoma	6
		Missing	58			Missing	50
**Lung squamous cell carcinoma**							
Male	368	Stage IA	89	Pharmacotherapy only	57	Squamous cell carcinoma, NOS	465
Female	130	Stage IB	150	Radiotherapy only	65	Basaloid	14
		Stage IIA	64	Both therapies	48	Keratinizing	13
		Stage IIB	94	No treatment	265	Papillary	3
		Stage IIIA	63	Missing	63	Large cell, nonkeratinizing	2
		Stage IIIB	18			Small cell, nonkeratinizing	1
		Stage IV	7				
		Stage I	3				
		Stage II	3				
		Stage III	3				
		Missing	4

### Selection of genes

**Figure 2:**
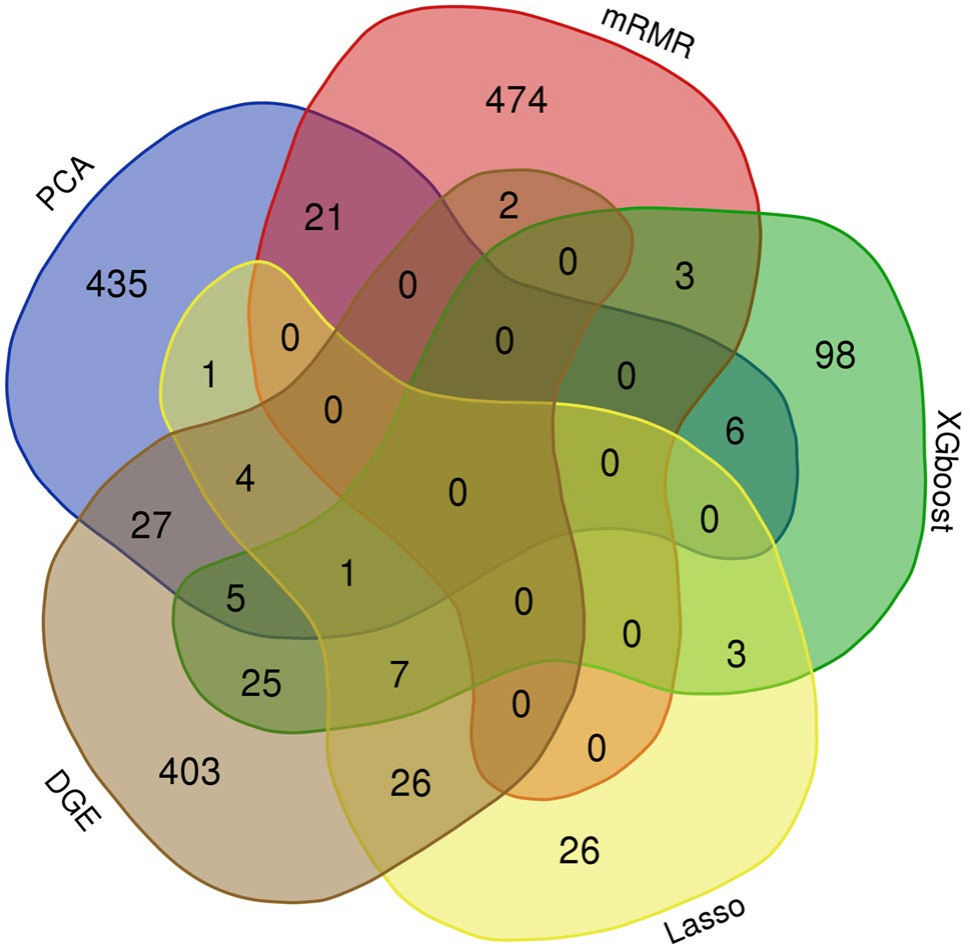
Venn diagram shows overlapping genes selected by each algorithm. Venn diagram of selected genes from PCA, mRMR, DGE, Lasso, and XGboost.

Top 500 genes from DGE (Table S1) were selected as top features based on their lowest p-values. Similarly, top 500 genes from the first principal component in PCA and the top 500 genes from mRMR (Table S1) were selected based on the ranking of the algorithm. Also, 148 genes in Xgboost (Table S1) and 68 genes in lasso (Table S1) using probability or prediction threshold of 0.5 were identified and selected. The different number of genes selected was due to the nature of the algorithm, with most of the parameters in each algorithm were set to default. The specifics of each metric can be found in the code at the data availability section Since each of these methods has its own selection criteria, the overlapping genes must satisfy multiple selection criteria, making them significant candidate biomarkers that differentiate LUAD and LUSC. Therefore, the five independent sets of top genes were compared with a Venn diagram to identify the overlapping genes detected by multiple algorithms. Venn diagram (Fig. 2) comparison detected 131 genes (Table S2) overlapped by two or more algorithms and 17 genes (Table 2) overlapped by three or more algorithms.

**Table 2:** 17 Biomarker candidate genes that were selected by three or more.

Genes	Upregulated or downregulated	Significantly expressed in LUSC or LUAD	Number of algorithms that selected the gene
KRT17 (Keratin 17)	Upregulated	LUSC	DGE, Lasso, PCA, XGBoost
KRT14 (Keratin 14)	Upregulated	LUSC	DGE, PCA, XGboost
KRT6A (Keratin 6A)	Upregulated	LUSC	DGE, PCA, XGboost
KRT5 (Keratin 5)	Upregulated	LUSC	DGE, PCA, XGboost
S100A2 (Calcium Binding Protein A2)	Upregulated	LUSC	DGE, PCA, XGboost
TUBA1C (Tubulin Alpha 1c)	Upregulated	LUSC	DGE, Lasso, XGboost
CELSR2 (Cadherin EGF LAG seven-pass G-type receptor 2)	Upregulated	LUSC	DGE, Lasso, XGboost
TRIM29 (Tripartite Motif Containing 29)	Upregulated	LUSC	DGE, Lasso, PCA
REPS1 (RALBP1 Associated Eps Domain Containing 1)	Upregulated	LUSC	DGE, Lasso, XGboost
PERP (P53 Apoptosis Effector Related To PMP22)	Upregulated	LUSC	DGE, Lasso, PCA
NECTIN1 (Nectin Cell Adhesion) Molecule 1	Upregulated	LUSC	DGE, Lasso XGboost
GPC1 (Glypican 1)	Upregulated	LUSC	DGE, PCA, XGBoost
MUC1 (Mucin 1, cell surface associated)	Downregulated	LUAD	DGE, Lasso, PCA
ELFN2 (Extracellular Leucine Rich Repeat And Fibronectin Type III Domain Containing 2)	Downregulated	LUAD	DGE, Lasso, XGboost
ARHGEF38 (Rho Guanine Nucleotide Exchange Factor 38)	Downregulated	LUAD	DGE, Lasso, XGboost
ARHGAP12 ( Rho GTPase Activating Protein 12)	Downregulated	LUAD	DGE, Lasso, XGboost
QSOX1 (Quiescin Sulfhydryl Oxidase 1)	Downregulated	LUAD	DGE, Lasso, PCA

### Validation of selected genes

**Figure 3:**
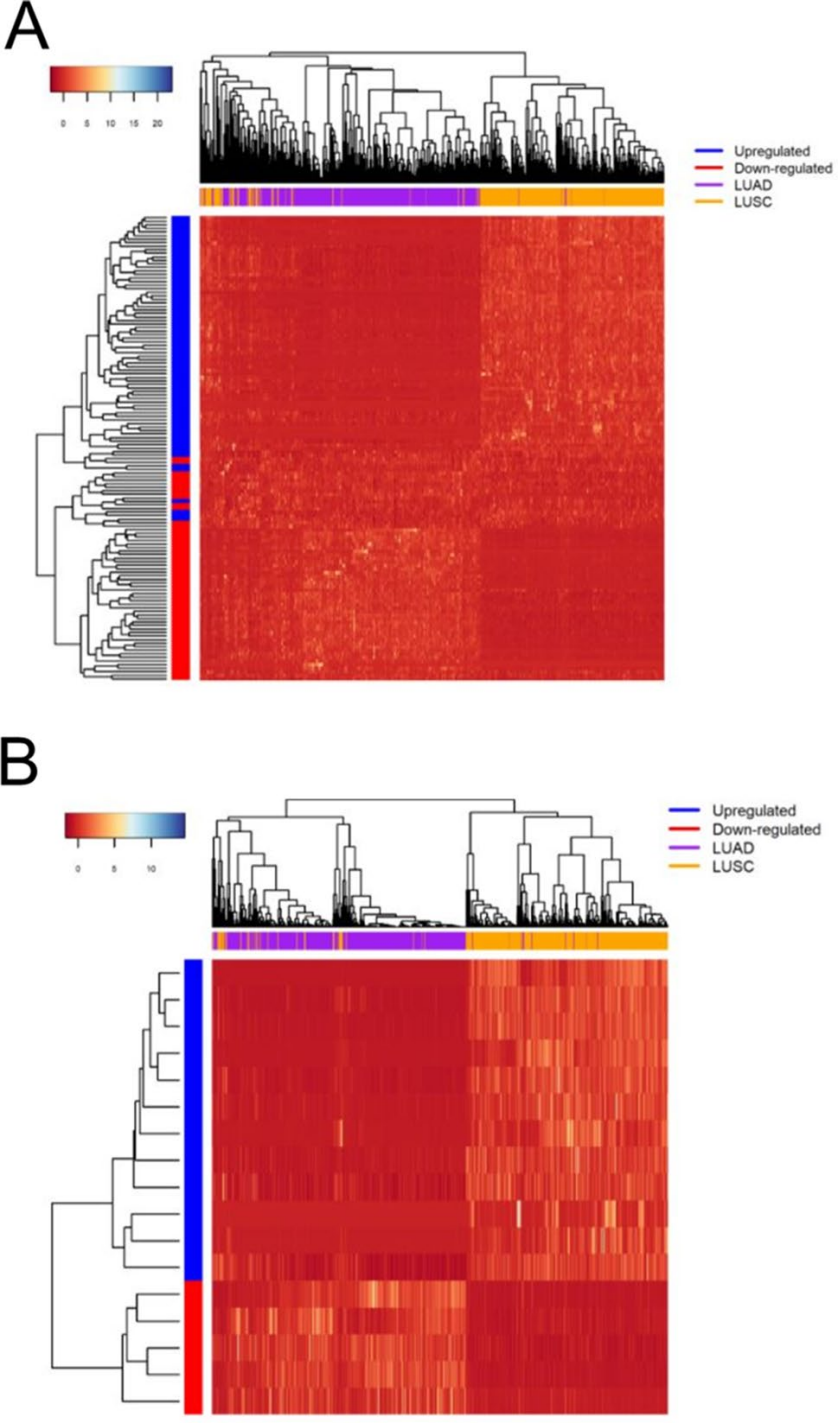
Heatmap shows the 131 selected genes (**A**) for gene expression analysis and the 17 selected genes (**B**) as biomarker candidates^87^. The x-axis represents the samples and the y-axis represents the genes.

**Figure 4:**
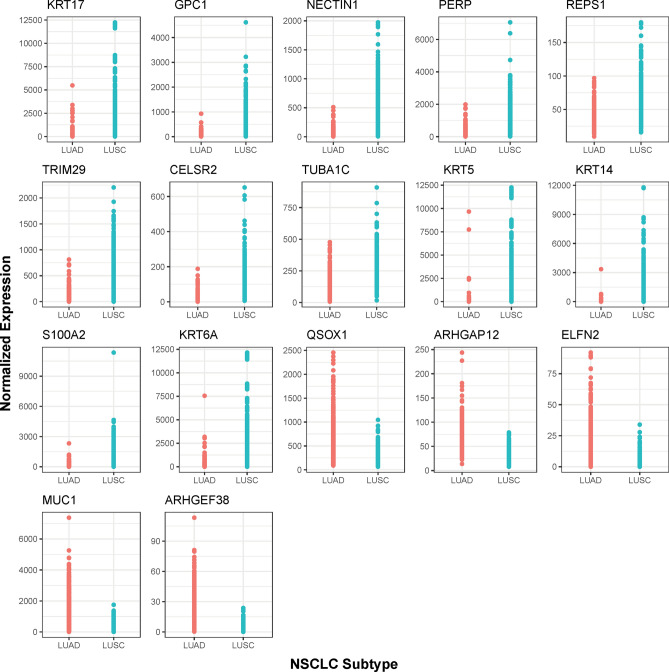
Normalized Gene Expression Distribution Dot Plots for the 17 Biomarker Candidates^87^. The x-axis represents the NSCLC subtypes and the y-axis represents the normalized gene expression values.

To evaluate how effective the selected genes are in classifying LUAD and LUSC, we used random forest to validate the top 500 genes selected from PCA, mRMR, and DGE, as well as the 148 genes from xgboost and 68 genes from lasso (Table S1). All of the validation results for each feature selection method returned high classification accuracies of over 90% (Table 3). To compare to the previous feature selection methods, the overlapping 131 genes were validated the same way as the other algorithms. The binary classification statistics (Table 3) were calculated using LUAD as ‘positive’ and LUSC as ‘negative’. The overlapping 131 genes showed comparable, if not better, results to the other algorithms (Table 3). The 17 proposed biomarkers also showed to be effective classifiers, having statistics comparable to the other algorithms despite only using 17 genes. Heatmaps for the top 131 and the top 17 genes were also generated (Fig. 3A,B). Both heatmaps, in particular the heatmap with 17 genes, displayed clear borders separating LUAD from LUSC. Dot plots of the gene expression distribution between LUAD and LUSC for each of the 17 genes are displayed in Fig. 4.

**Table 3:** LUAD and LUSC Classification Statistics.

Feature selection method	Accuracy	Specificity	Sensitivity	Precision	F-measure	95% Bootstrap confidence interval
DGE (Top 500)	0.932476	0.901235	0.966443	0.9	0.932039	(0.9035, 0.9614)
PCA (Top 500)	0.942122	0.901235	0.986577	0.90184	0.942308	(0.9132, 0.9678)
mRMR (Top 500)	0.916399	0.888889	0.946309	0.886792	0.915584	(0.8842, 0.9453)
Lasso (68 Genes)	0.938907	0.907407	0.973154	0.90625	0.938511	(0.9100, 0.9646)
Xgboost (148 Genes)	0.935691	0.901235	0.973154	0.900621	0.935484	(0.9068, 0.9614)
Overlapping 131 Genes	0.938907	0.895062	0.986577	0.896341	0.939297	(0.9100, 0.9646)
17 Proposed Biomarkers	0.92926	0.889	0.9735	0.88957	0.9296	( 0.9003, 0.9550 )

### Identification of the 17 potential biomarkers and their ROC analysis

**Figure 5:**
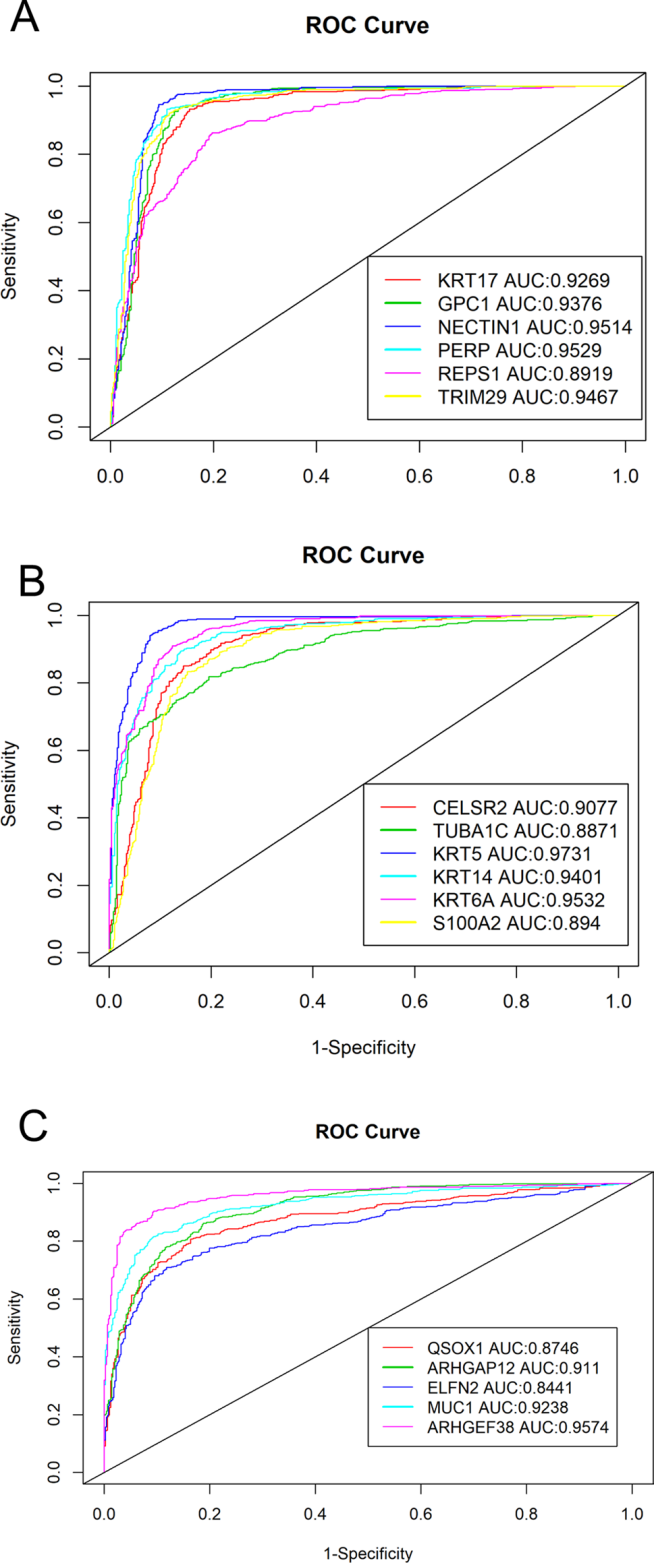
ROC and AUC analysis demonstrate discriminating potential for Upregulated (**a**,**b**) and Downregulated (**c**) Genes in TCGA Dataset^87^. X-axis is sensitivity, or true positive rate (TPR). The Y-axis is 1-Specificity, or false positive rate (FPR). Higher AUC indicates higher discriminating potential for the gene.

The 17 biomarker candidates (Table 2) were subjected to ROC curve analysis (Fig. 5). Most of the genes had areas under the curve (AUC) of over 0.9, with NECTIN1 (0.9514), PERP (0.9529), KRT5 (0.9731), KRT6A (0.9532), and ARHGEF38 (0.9574) having AUC of over 0.95. Among the upregulated genes (Fig. 5A), KRT5 has the highest AUC of 0.9731, thereby displaying the most significant diagnostic potential in classifying LUAD and LUSC, consistent with the study reported by Jain Xiao et al.^6^ in which KRT5 also had the highest diagnostic potential. All of the upregulated genes show significant discrimination potential as well (Fig. 5A,B).

**Figure 6:**
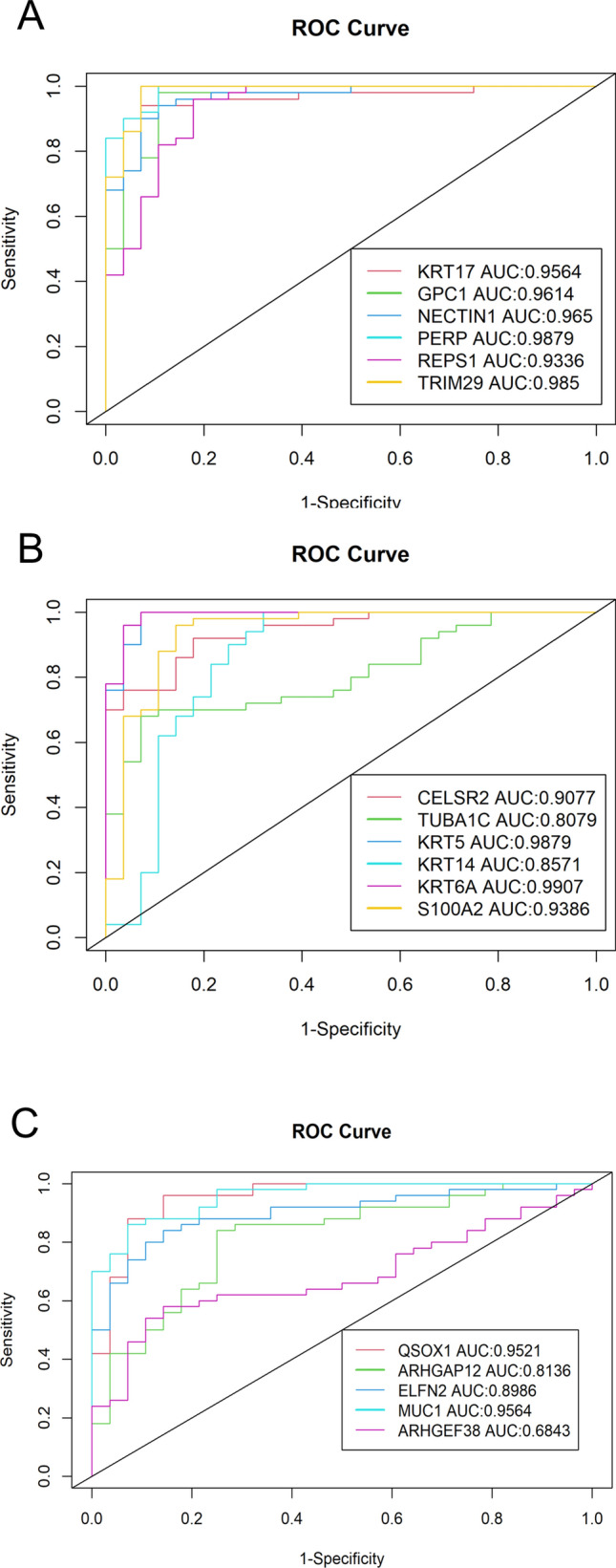
GSE28582 microarray dataset ROC and AUC validation of the 17 candidate biomarkers^87^. (**A**,**B**) The upregulated genes, and (**C**) shows the downregulated genes. The x-axis represents sensitivity, or true positive rate (TPR). The y-axis is 1 − Specificity, or false positive rate (FPR). Higher AUC indicates higher discriminating potential for the gene.

To minimize the inherent RNA expression noise and to ensure that these results are reproducible, an external dataset GSE28582 was used for external validation. AUC and ROC were also used to analyze the 17 genes in GSE28582 validation dataset (Fig. 6). Largely consistent with our result, most of the genes show AUC values well above 0.9; all except one gene, ARHGEF38, have AUC values above 0.8 (Fig. 6).

### Kaplan Meier plotter analysis of the 17 potential biomarkers

Of the 17 potential biomarkers (Table 2), only CELSR2 shows a significant prognostic p-value in LUSC, with its higher expression corresponding to a more favorable prognosis in LUSC (Table 4). In contrast, many genes show significant prognostic potential in LUAD. High expressions of KRT17, KRT6A, S100A2, TRIM29, REPS1, and GPC1 correspond to an unfavorable prognosis in LUAD, while high expressions of PERP, ELFN2, ARHGAP12, and QSOX1 correspond to a favorable prognosis in LUAD (Table 4).

**Table 4:** Kaplan Meier prognostic values of the 17 biomarker.

	LUAD		LUSC	
	HR (95% CIs)	P-value/FDR	HR (95% CIs)	P-value/FDR
KRT17	1.28 (1.01–1.61)	0.037/0.0629	1.11 (0.88–1.4)	0.39/0.947
KRT14 (EBS4)	1.19 (0.94–1.5)	0.14/0.2164	1.2 (0.95–1.52)	0.13/1
KRT6A (K6C)	1.67 (1.32–2.12)	1.6e−05/0.00014	0.99 (0.78–1.25)	0.92/0.98
KRT5	1.14 (0.9–1.43)	0.28/0.366	1 (0.79–1.27)	1/1
S100A2	1.73 (1.36–2.19)	4.3e−06/7.31E−5	1.07 (0.85–1.36)	0.55/1
TUBA1C	1.1 (0.87–1.39)	0.43/0.522	1.2 (0.94–1.52)	0.14/0.793
CELSR2	0.92 (0.73–1.16)	0.47/0.533	0.79 (0.62–1)	0.049/0.833
TRIM29	1.31 (1.04–1.66)	0.022/0.0416	0.93 (0.74–1.18)	0.57/0.969
REPS1	1.38 (1.08–1.76)	0.0093/0.0226	0.9 (0.66–1.23)	0.51/1
PERP	0.67 (0.52–0.85)	0.0012/0.0051	0.85 (0.62–1.16)	0.3/0.85
NECTIN1 (PVRL1)	1.19 (0.94–1.5)	0.14/0.198	0.94 (0.74–1.2)	0.63/0.974
GPC1	1.36 (1.08–1.72)	0.0091/0.0258	0.98 (0.77–1.23)	0.83/1
MUC1	1.02 (0.81–1.29)	0.84/0.084	1.02 (0.8–1.29)	0.88/1
ELFN2	0.72 (0.56–0.92)	0.0076/0.02584	1.07 (0.78–1.47)	0.67/0.876
ARHGEF38 (FLJ20184)	0.97 (0.77–1.23)	0.83/0.882	1.16 (0.91–1.47)	0.22/0.748
ARHGAP12	0.61 (0.48–0.77)	2.3e−05/0.00013	1.17 (0.93–1.49)	0.18/0.765
QSOX1	0.76 (0.6–0.96)	0.021/0.0446	0.95 (0.75–1.2)	0.66/0.935

### GO term enrichment analysis

To further understand the biological differences between LUAD and LUSC, we performed gene expression analysis by splitting the identified 131 genes into two groups: 57 downregulated and 74 upregulated genes in LUSC compared to LUAD. Functional pathway annotation of these two groups of genes was performed using The Database for Annotation, Visualization and Integrated Discovery (DAVID)^27^ analysis tool with Gene Ontology (GO) biological pathway enrichments. GO terms with P-value < 0.01 were obtained (Tables S3 and S4). The top 10 most significantly upregulated and downregulated GO terms ranked by p-value are shown in Table 5. In addition, DAVID has the functionality to group similar GO terms into clusters of the same biological pathway. To elucidate the potential biological differences between LUAD and LUSC, the top five most significantly upregulated and downregulated clusters ranked by enrichment scores were determined (Table 6 and Tables S5 and S6).

**Table 5:** Top 10 Upregulated and Downregulated GO Biological Pathways.

Top 10 upregulated pathways			Top 10 downregulated pathways		
GO term	Pathway	P-value	GO term	Pathway	P-value
GO:0009888	Tissue development	4.45E−07	GO:0002576	Platelet degranulation	2.86E−04
GO:0045104	Intermediate filament cytoskeleton organization	8.82E−07	GO:1901575	Organic substance catabolic process	8.18E−03
GO:0045103	Intermediate filament-based process	9.95E-−07	GO:0009057	Macromolecule catabolic process	8.29E−03
GO:0007155	Cell adhesion	4.25E−06	GO:0045055	Regulated exocytosis	1.05E−02
GO:0022610	Biological adhesion	4.49E−06	GO:0009056	Catabolic process	1.32E−02
GO:0008544	Epidermis development	4.64E−06	GO:00034613	Cellular protein localization	1.80E−02
GO:0098609	Cell–cell adhesion	5.07E−06	GO:0070727	Cellular macromolecule localization	1.89E−02
GO:0034330	Cell junction organization	9.93E−06	GO:0043129	Surfactant homeostasis	2.36E−02
GO:2001233	Regulation of apoptotic signaling pathway	3.06E−05	GO:0016553	Base conversion or substitution editing	2.65E−02
GO:0061436	Establishment of skin barrier	5.65E−05	GO:0048875	Chemical homeostasis within a tissue	2.94E−02

**Table 6:** Top 5 Clusters of Upregulated and Downregulated Biological pathways.

Top 5 clusters of upregulated biological pathways		Top 5 clusters of downregulated biological pathways	
Cluster	Enrichment score	Cluster	Enrichment score
Cell adhesion	4.05	Platelet degranulation and exocytosis	1.34
Intermediate filament organization	3.87	Tyrosine kinase pathways	0.74
Cell junction organization	3.42	Homeostasis	0.69
Cell component organization	3.28	Protein translation and localization	0.68
Hemidesmosome assembly	2.67	Circulatory system regulation	0.63

In the upregulated group, most pathways are concentrated in cell adhesion, intermediate filament organization, and cell junction assembly. In the downregulated group, the most significant cluster is platelet degranulation and cell exocytosis, as well as other pathways such as tyrosine kinase signaling pathway, homeostasis, protein translation and circulatory system. These results suggest that LUSC tends to express more genes related to cell adhesion and cytoskeleton organization, and LUAD tends to express more genes involved in platelet degranulation and exocytosis, along with other signaling pathways.

### Reactome gene expression analysis

**Figure 7:**
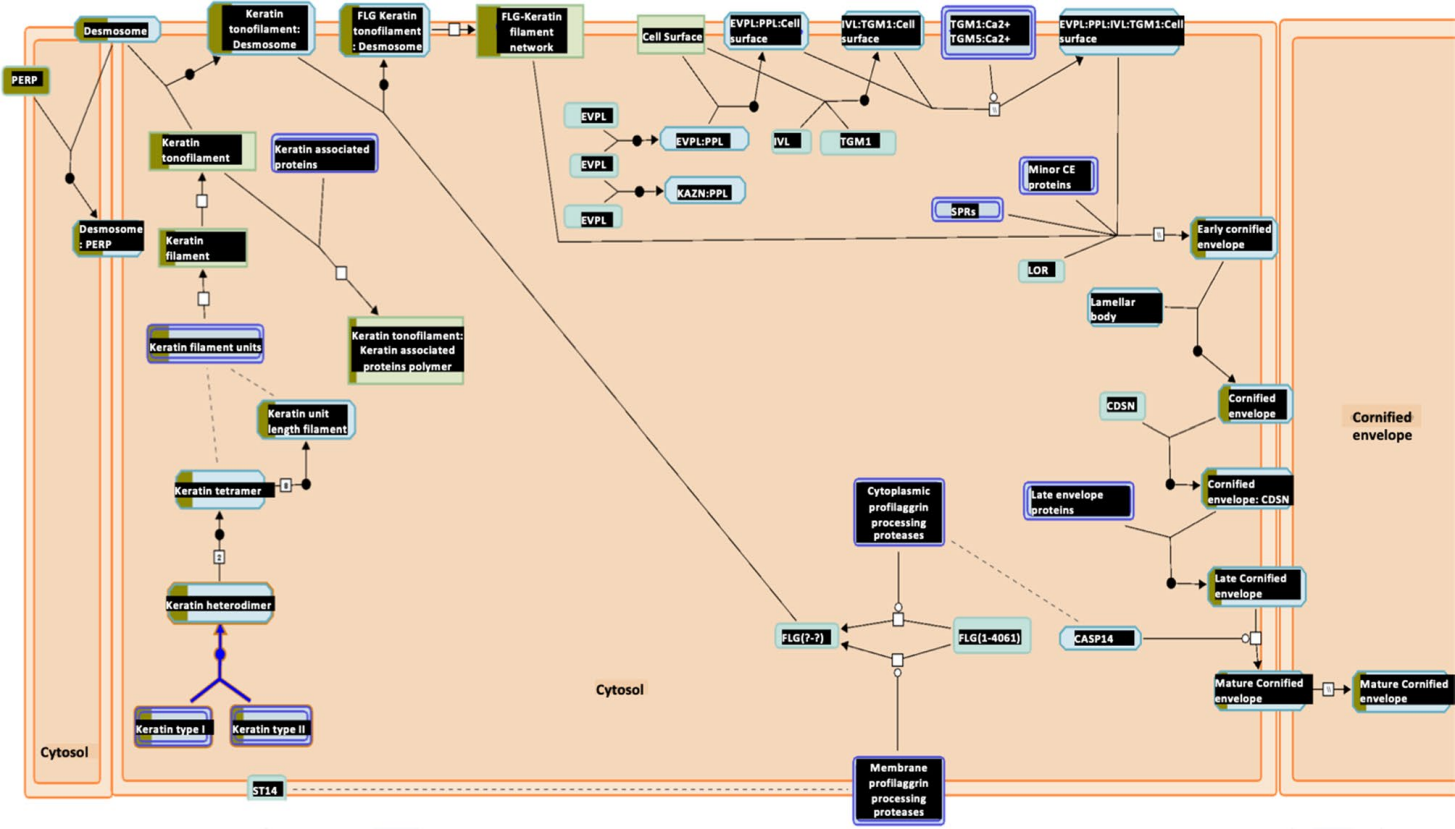
Keratinization pathway is upregulated in LUSC^28^. The Keratinization pathway is the most upregulated pathway according to Reactome analysis with p-value 3.33E−15 and FDR 1.95E−12. The boxes partially highlighted in brown indicate the number of genes identified in the analysis that are associated with each box.

**Figure 8:**
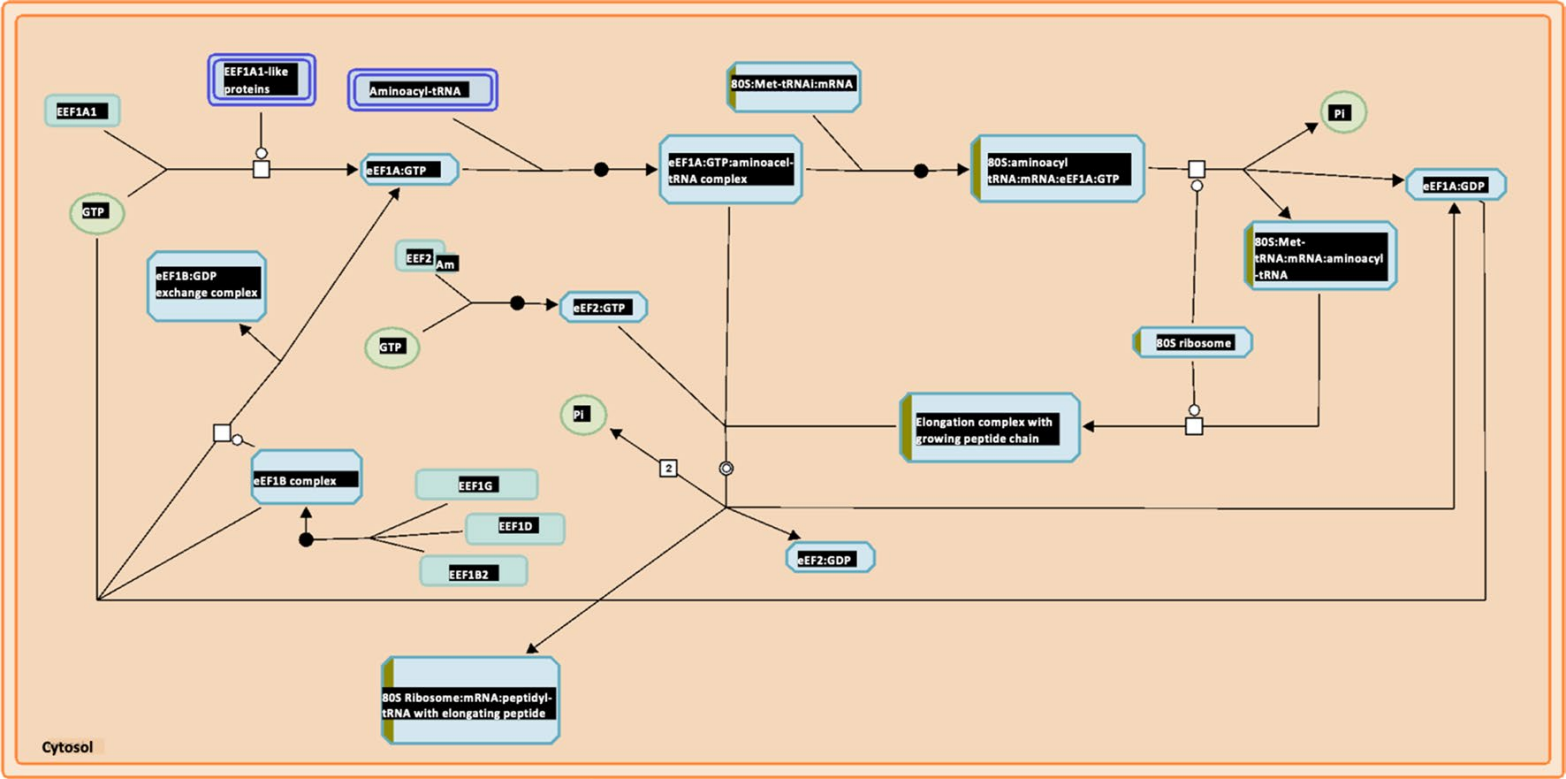
Peptide elongation pathway is downregulated in LUSC when compared to LUAD^28^. The peptide elongation pathway is the most down-regulated pathway according to Reactome analysis with p-value 9.72E−6 and FDR 0.00157. The boxes partially highlighted in brown indicate the number of genes identified in the analysis that are associated with each box.

Reactome pathways^28^ were also generated for both upregulated and downregulated groups. The most significantly upregulated pathway is the cornification, or the keratinization pathway (Fig. 7, Table S7), along with other similar pathways related to cell adhesion, which is consistent with GO term analysis. TP53 regulation pathway, which is often implicated in cancer, is among the top enriched pathways as well (Table S7). For the downregulated group, the most significant pathway is peptide elongation synthesis (Fig. 8, Table S8), which GO term analysis also reveals to be significant.

### KEGG gene expression analysis

Only the p53 signaling pathway appeared in the upregulated group (Table 7) in Kyoto Encyclopedia of Genes and Genomes (KEGG)^29^ gene expression analysis. Though it has a p-value of slightly over 0.01, this result is consistent with Reactome analysis which ranks TP53 regulation as the second most upregulated pathway after keratinization and other cell junction related pathways. Only the lysosome seems to be significant in the downregulated group (Table 7). The lysosomal pathway is coherent with platelet degranulation and exocytosis, as reported in GO term analysis. Even though the ribosomal pathway has a p-value slightly greater than 0.05, it is most likely important as it is also shown to be significantly enriched in both GO and Reactome term analyses (Tables S3 and S8).

**Table 7:** KEGG Upregulated and Downregulated Pathways.

KEGG upregulated pathways			KEGG downregulated pathways		
KEGG term	Pathway	P-value	KEGG term	Pathway	P-value
Hsa04115	P53 signaling pathway	0.0476	Hsa04142	Lysosome	0.00727
NA	NA	NA	Hsa03010	Ribosome	0.0749

## Discussion

Previous studies have utilized traditional feature selection and machine learning methods for cancer diagnosis, detection, and classification^10,11,22^, but few have extended them to study potential biomarkers and biological pathways to discriminate between LUAD and LUSC. To improve cancer classification accuracy, novel machine learning, and feature selection methods have been developed^12,30–32^. However, few studies have used overlapping features from different methods for classification, gene expression analysis, and biomarker discovery. To provide a proof of concept for the validity of this method, we took advantage of the capabilities and the strengths of PCA, mRMR, XGboost, DGE, and lasso to select 131 overlapping genes for classification and gene expression analysis, and 17 genes for classification and potential biomarkers. Overall, the overlapping 131 genes showed several high-ranking metrics with lasso and PCA methods. Though the best method may vary depending on the metric, the classification result of using both the overlapping 131 and 17 genes was by many metrics comparable if not better than the other methods that use more genes. The 131 overlapped genes achieved the highest sensitivity with PCA, the second highest accuracy with lasso, and the second highest F-measure overall, indicating that overlapping feature selection methods can be used to perform cancer classification.

Furthermore this method proves to be valuable in biomarker discovery. In agreement with our result, previous studies have reported levels of several genes to be greatly elevated in LUSC compared to LUAD; these genes include KRT6^6,8,33,34^, KRT5^6,8,35^, KRT14^8,33,34^, KRT17^8,33^, PERP^8,33^, TRIM29^8,33^, GPC1^8^, CELSR2^8^, S100A2^8^, and TUBA1C^36^. Also, consistent with our result, levels of QSOX1^33^ and MUC1^8^ were reported to be lower in LUSC than in LUAD. Many current biomarkers such as Tumor Protein P63 (TP63), Napsin A Aspartic Peptidase (NAPSA), Melanophilin (MLPH), Desmocollin 3 (DSC3), and others are also part of the top 131 genes selected by our method^33,37–40^. To our knowledge, ARHGAP12, ARHGEF38, ELFN2, NECTIN1, and REPS1 are among the top 17 genes in this study to be identified as biomarkers for the first time. All 17 candidate biomarkers, except ARHGEF38, are also validated in GSE28582 exhibiting high discriminating potential. Although the selection of ARHGEF38 may be due to bias in the TCGA dataset, it is important to note that there are many more samples in TCGA compared to GSE28582; GSE28582 as a microarray dataset is also significantly worse than RNAseq at detecting gene expression differences when the expression values are low or when the fold change is less than 2^41–43^. Notably, ARHGEF38 has relatively lower fold change and expression value.

Moreover, studies have shown that biomarkers for diagnosis and prognosis are most reliable when they are biologically related to the disease in addition to being statistically significant^44,45^. Although this study is primarily data-driven, the results reveal biomarkers that would corroborate with a knowledge-based approach. For instance, the most significant candidate biomarkers between LUAD and LUSC are all cytokeratins and cadherins, which is reasonable because they are markers of squamous epithelial cells. In particular, NECTIN1, as a novel cadherin biomarker, consistently demonstrates high discriminating potential both in the TCGA and the external validation dataset; it also directly binds and signals fibroblast growth factor receptor^46^, a pathological signaling pathway that is more prominent in LUSC^47,48^. NECTIN1 also serves a key role in herpes simplex virus type 1 (HSV-1) viral entry and is important in oncolytic therapy in squamous cell carcinomas^49,50^. Similarly, it is logical that MUC1 can be used to identify LUAD, as it is a marker for columnar cells from which LUAD arise. In addition to satisfying the aims of both data-driven and knowledge-based approach, many of the 17 genes identified through this method show significant prognostic importance, particularly in LUAD (Table 4).

The other candidate biomarkers also show strong association with cancers. ARHGEF38 and ARHGAP12 are both part of the Rho family GTPase regulators. Rho GTPases are essential to cell cytoskeletal structure, motility, and morphogenesis, and they have been implicated in many cancer proliferation and metastases^51–54^. The other upregulated genes ELFN2, QSOX1, and MUC1 have been shown to directly promote metastasis in various cancers^55–59^, including lung cancer. Furthermore, the loss of certain genes upregulated in LUSC such as TRIM29 and KRT6A is associated with more cellular invasion^60,61^. Clinical differences between LUAD and LUSC are well known. In particular, LUAD has a higher metastatic rate than LUSC^62^. Studying these potential biomarkers may provide insight into tumor progression, metastatic, and therapeutic differences between LUAD and LUSC. Overall, these results not only align with known literature, but also provide reasonable and promising biomarkers, suggesting that using overlapping feature selection methods can be used to reliably detect new biomarkers. With the validity of this overlapping method shown both in cancer classification and biomarker identification, we performed gene expression analysis for further investigation.

Aside from cell adhesion or cytoskeleton organization, LUSC demonstrates higher regulation of p53 signaling in both KEGG and Reactome analyses. It is known that TP53 mutation is more common in LUSC than in LUAD^63–65^, and that such mutation may predominantly be a non-truncated mutation in LUSC leading to higher expression levels of genes involved in the p53 regulation pathway^66^. Moreover, P53 mutations often lose their tumor suppression function while gaining oncogenic abilities, leading to increased cell growth and proliferation compared to LUAD^67^.

The most prominent pathway associated with LUAD, compared to LUSC, is platelet degranulation and exocytosis (Tables 5, 6). Interestingly, lung cancer is the most common malignancy to coexist with venous thromboembolism, especially pulmonary embolism^68^. LUAD, in particular, has been shown to be an independent risk factor for pulmonary embolism even among lung cancers^69,70^. Because platelet granulation directly causes thrombus formation, the differential enrichment of platelet granulation pathway can therefore help explain a more active and a more common hypercoagulation and thrombotic process in LUAD compared to LUSC^71^. In addition, platelet degranulation can modulate innate immunity via the release of cytokines, and platelet-leukocyte interactions can lead to leukocyte recruitment and activation in cancer^72^. In fact, CD63, one of the genes in the platelet degranulation pathway (Tables S3 and S6), is directly involved in leukocyte recruitment through endothelial P-selectin^73^. LUSC has recently been associated with a relatively more suppressed immune response, implying a more active immune response in LUAD, which supports our result^67,74^.

There are several limitations of this study. One of them is that this study does not prioritize the RNA expression fold changes, which some groups have used to rank differentially expressed genes^75,76^. Also, although this study aims to minimize the discovery of false positive biomarkers by overlapping different feature selection methods, the proposed biomarker candidates in this study still lack experimental verification. Nevertheless, these results may shed light into the biological differences between LUAD and LUSC, as well as aid the discovery of better diagnosis and treatment for each^77,78^.

In conclusion, we designed and implemented a workflow of overlapping five different feature selection methods to perform cancer classification, identify novel biomarkers, and study biological differences in NSCLC. This overlapping method proves to be reliable in both cancer classification and biomarker identification, yielding statistically promising genes that also support our current knowledge. We identified ARHGAP12, ARHGEF38, ELFN2, NECTIN1, and REPS1 as novel biomarkers, along with 12 other strong biomarker candidates. We also provided insight into potential explanations for different clinical findings and biological characteristics between LUSC and LUAD through gene expression analysis. Further validation studies of these biomarkers and biological mechanisms are therefore warranted.

## Method

### RNA-Seq data processing

The LUAD and LUSC HTSeq read counts data were downloaded from TCGA^13^ using TCGAbiolinks from R^79,80^. As of June 2020, there were 529 LUAD and 498 LUSC samples. The samples were normalized using TMM method and standardized using the CPM (read counts per million) function in R. Genes < 1 CPM in over 600 samples were considered noise and discarded to obtain 14,010 genes. The filtered genes were analyzed with different gene selection methods to further narrow down potential gene candidates for biomarkers and pathway analyses.

### Gene selection and cancer classification

Gene selection analysis was performed using five different selection methods to generate five independent sets of top genes (Fig. 1). The 5 independent sets were compared, and the resulting overlapped genes were used for cancer classification, biomarker identification, and gene expression analysis. The selection methods used were DGE, PCA, xgboost, lasso, and mRMR. DGE between LUAD and LUSC was performed using the edgeR package^81^. Though there are other options to perform differential gene expression analysis, edgeR was chosen mostly because of its speed and efficiency in analysis. Also, one of the other popular algorithm, DESeq, has also been shown to yield similar result as edgeR^16^. After using edgeR analysis and filtering for genes that have FDR < 5E−2 and log(Fold Change) > 0.5, 4702 genes were identified as differentially expressed. Top 500 of the 4702 differentially expressed genes (Table S1) were selected as top features based on their lowest p-values; validation of these genes was performed using random forest with the ranger package^82^. The top 500 genes from the first principle component in PCA and the top 500 genes ranked from mRMR^83^ algorithm were selected and validated the same way as the differentially expressed genes. Genes with probability or prediction threshold over 0.5 were selected from Xgboost^84^ and lasso^85^ (Table S1), and validated in a similar manner as the other algorithms. For each validation, the data were randomly split into a training set and a testing set in a 7:3 ratio, where the training set was used to construct the model while the testing set was used to evaluate the model’s performance. To compare each selection method more effectively, we split the training sets and testing sets the same way for all validations. We applied fivefold cross validation to decide the optimal parameters for each training model and estimated its accuracy by applying the best determined parameters to the test set. The detailed parameters can be found in the data availability section.

For classification and gene expression analysis, we selected genes that were detected by at least two methods, and they were validated using ranger^82^. We also used bootstrapping^86^ with 10,000 replicates to calculate the confidence interval for the accuracy of each method, including the proposed method of classification. The genes that were detected by at least 3 methods were considered candidate biomarkers. Their diagnostic potential was determined and assessed using receiving operating characteristics (ROC) curve analysis. GSE28582^24,25^, was used as an external dataset to validate the chosen 17-gene classifier.

### Prognostic value analysis using Kaplan–Meier plotter

Kaplan–Meier Plotter is an online database that contains comprehensive clinical and microarray data for various cancers, including lung cancer^26^. Prognostic values of the identified biomarkers in LUAD and LUSC were evaluated using Kaplan–Meier Plotter with each gene used as an univariate analysis. The parameters were set such that the only restricted subtypes were LUAD and LUSC, and the median was used as the cutoff. The rest of the parameters were in the default settings.

### Gene expression analysis of selected genes

To further investigate and understand the biological difference between LUAD and LUSC, we performed pathway enrichment analysis using KEGG^29^, Gene Ontology (GO), and Reactome^28^. Modified Fisher’s exact tests were performed using DAVID v6.8^27^. Pathways with false discovery rate (FDR) < 5% or p-value less than 0.01 were considered significant. These databases were all accessed in November 2020.

## Supplementary Information

Supplementary Tables.

## Data Availability

All data generated and/or analyzed during the current study are included in this published article (and its supplementary information files). The custom code used for data analysis can be accessed at https://github.com/chenjoe569/NSCLC-Research.

## Abbreviations

AUC: Area under curve
DAVID: The Database for Annotation, Visualization, and Integrated Discovery
DGE: Differential gene expression
FPR: False positive rate
GO: Gene ontology
KEGG: Kyoto Encyclopedia of Genes and Genomes
Lasso: Least absolute shrinkage and selection operator
LUAD: Lung adenocarcinoma
LUSC: Lung squamous cell carcinoma
mRMR: Minimum redundancy maximum relevance
NSCLC: Non-small cell lung cancer
PCA: Principal component analysis
ROC: Receiving operating characteristics
TCGA: The Cancer Genome Atlas
TPR: True positive rate
Xgboost: Extreme gradient boosting
QSOX1: Quiescin sulfhydryl oxidase 1
ARHGAP12: Rho GTPase activating protein 12
ARHGEF38: Rho guanine nucleotide exchange factor 38
ELFN2: Extracellular leucine rich repeat and fibronectin type III domain containing 2
MUC1: Mucin 1, cell surface associated
GPC1: Glypican 1 GPC1
NECTIN1: Nectin cell adhesion molecule 1
PERP: P53 apoptosis effector related to PMP22
REPS1: RALBP1 associated Eps domain containing 1
TRIM29: Tripartite motif containing 29
CELSR2: Cadherin EGF LAG seven-pass G-type receptor 2
TUBA1C: Tubulin alpha 1c
S100A2: S100 calcium binding protein A2
KRT5: Keratin 5
KRT14: Keratin 14
KRT6A: Keratin 6A
TP63: Tumor protein P63
NAPSA: Napsin A aspartic peptidase
MLPH: Melanophilin
DSC3: Desmocollin 3
